# Targeting Sphingosine Kinase 1 in Carcinoma Cells Decreases Proliferation and Survival by Compromising PKC Activity and Cytokinesis

**DOI:** 10.1371/journal.pone.0039209

**Published:** 2012-06-25

**Authors:** Nataliya Kotelevets, Doriano Fabbro, Andrea Huwiler, Uwe Zangemeister-Wittke

**Affiliations:** 1 Institute of Pharmacology, University of Bern, Bern, Switzerland; 2 Novartis Pharma AG, NIBR Basel, Switzerland; 3 Pharmazentrum Frankfurt/ZAFES, University Hospital Frankfurt, Frankfurt am Main, Germany; 4 Institute of Biochemistry, University of Zürich, Zürich, Switzerland; Wayne State University School of Medicine, United States of America

## Abstract

Sphingosine kinases (SK) catalyze the phosphorylation of proapoptotic sphingosine to the prosurvival factor sphingosine 1-phosphate (S1P), thereby promoting oncogenic processes. Breast (MDA-MB-231), lung (NCI-H358), and colon (HCT 116) carcinoma cells were transduced with shRNA to downregulate SK-1 expression or treated with a pharmacologic SK-1 inhibitor. The effects of SK-1 targeting were investigated by measuring the level of intracellular sphingosine, the activity of protein kinase C (PKC) and cell cycle regulators, and the mitotic index. Functional assays included measurement of cell proliferation, colony formation, apoptosis, and cell cycle analysis. Downregulation of SK-1 or its pharmacologic inhibition increased intracellular sphingosine and decreased PKC activity as shown by reduced phosphorylation of PKC substrates. In MDA-MB-231 cells this effect was most pronounced and reduced cell proliferation and colony formation, which could be mimicked using exogenous sphingosine or the PKC inhibitor RO 31-8220. SK-1 downregulation in MDA-MB-231 cells increased the number of cells with 4N and 8N DNA content, and similar effects were observed upon treatment with sphingosine or inhibitors of SK-1 or PKC. Examination of cell cycle regulators unveiled decreased cdc2 activity and expression of Chk1, which may compromise spindle checkpoint function and cytokinesis. Indeed, SK-1 kd cells entered mitosis but failed to divide, and in the presence of taxol also failed to sustain mitotic arrest, resulting in further increased endoreduplication and apoptosis. Our findings delineate an intriguing link between SK-1, PKC and components of the cell cycle machinery, which underlines the significance of SK-1 as a target for cancer therapy.

## Introduction

The cellular sphingolipid signaling pathway is a highly conserved balanced system comprising ceramide and sphingosine with proapoptotic functions on the one hand, and sphingosine-1-phosphate (S1P) promoting cell survival and proliferation on the other hand [1]. Elevated S1P favors tumor development and progression as it inhibits apoptosis and promotes cell proliferation, survival and migration. The level of intracellular S1P is maintained by sphingosine kinase 1 (SK-1) and the less well defined isoform sphingosine kinase 2 (SK-2) [2], [3]. SK-1 is overexpressed in many human tumors [4], [5] where it contributes to malignant progression and thus represents a promising molecular target for cancer therapy [6].

The recent finding that SK-1, SK-2 and the S1P_5_ receptor show centrosomal localization in cells has raised speculations about a direct role of the kinases and their metabolites in cell cycle regulation [7]. Whereas ceramide was shown to inhibit proliferation by inducing cell cycle arrest at the G0/G1 boundary [8], [9], sphingosine inhibits proliferation by inhibition of PKC [10], [11]. Interestingly, other studies also demonstrated that glycosphingolipids may function as inhibitors or stimulators of PKC activity [12], though the molecular details underlying this differential effects are still unclear. For sphingosine, biochemical analysis unveiled that it inhibits PKC by preventing the binding of phorbol esters without affecting the catalytic domain [13].

The serine/threonine kinase PKC is the major cellular target of tumor promoting phorbol esters and thus considered crucial for carcinogenesis [14]. It consists of a family of isoenzymes which are divided into three classes based on their activation requirements [15]. PKCs induce a variety of fundamental biological effects including cell proliferation and differentiation, membrane transport and gene transcription. Many of these effects drive malignant progression and result either directly from activated PKC or indirectly through its downstream effectors [16] **.** Consequently, PKC has been investigated as a target for cancer therapy, and a myriad of drugs from small molecule inhibitors to antisense oligonucleotides has been evaluated for therapeutic efficacy in preclinical tumor models and oncology trials [17]. Members of the PKC family were also found to be involved in cell cycle regulation by interfering in checkpoint functions [18], [19].

The cell cycle is controlled by sequential activation of cyclin-dependent kinases and their cyclin substrates. p34cdc2, also named Cdk1, together with cyclin B1 constitutes the mitosis promoting factor (MPF) which is crucial for G2/M transition and execution of mitosis [20]–[22]. Interestingly, both subunits of the MPF can be phosphorylated by PKC and PKC inhibitors may thus indirectly affect cdc2 activity too [23]. The DNA damage effector checkpoint kinase 1 (Chk1) is another multifunctional kinase of the cell cycle machinery which regulates the DNA damage response and is further required for spindle checkpoint function during early mitosis and for cytokinesis before cell division is completed [24]. In mitotic cells abrogation of Chk1 causes the typical defects including chromosome misalignment, cytokinetic regression and endoreduplication [25].

The therapeutic potential of targeting SK-1 and its product S1P in cancer has been reported in various preclinical studies [2]. We previously demonstrated that downregulation of SK-1 in carcinoma cells induced oxidative stress, which facilitated apoptosis induced by DNA damaging agents [26]. Here, we demonstrate that in carcinoma cells of various histotypes loss of SK-1 activity by genetic or pharmacologic inhibition compromises PKC due to intracellular sphingosine accumulation and establish a link between SK-1, PKC and M phase-specific cell cycle regulators, which may help improving our understanding of sphingolipid signaling in cancer.

## Results

### SK-1 Knockdown Results in Sphingosine Accumulation

**Figure 1:**
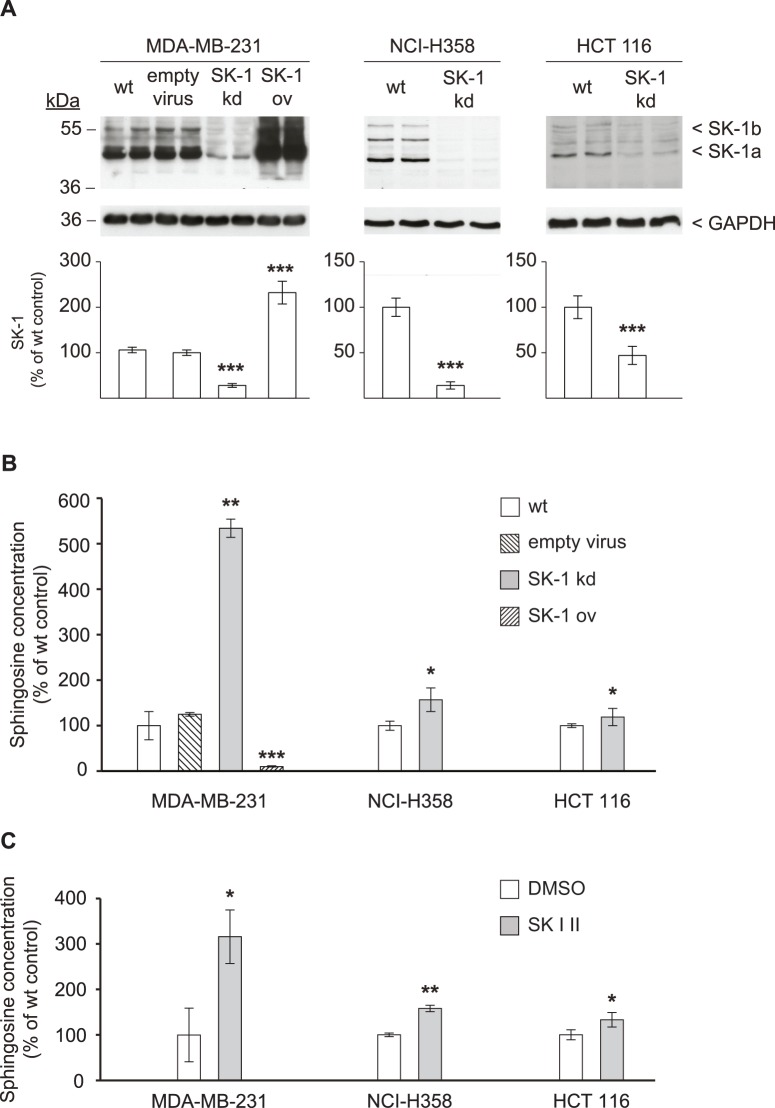
Downregulation of SK-1 results in sphingosine accumulation. (A) MDA-MB-231, NCI-H358, and HCT 116 cells were transduced with lentiviral SK-1 shRNA to downregulate SK-1 expression (SK-1 kd) or left untreated (wild-type, wt). MDA-MB-231 cells transduced with empty virus or transfected with the SK-1 cDNA for overexpression (SK-1 ov) are shown for comparison. Cell lysates were prepared and SK-1 protein expression was detected by Western blotting using antibodies against SK-1 (dilution 1∶1000) or GAPDH (dilution 1∶2000) as loading control. The two SK-1 splice variants SK-1a and SK-1b run at 43 and 51 kDa, respectively. The films were digitized and for each protein lane a density blot was measured. Each value in the graph represents the mean ± SD band density for each group (*n = *3); ***p<0.001 compared to wt cells. (B) cellular sphingosine was determined in the genetically modified SK-1 kd carcinoma cell lines, in the respective wt cells, and additional MDA-MB-231 control cells as described above (empty virus transfected, SK-1 ov). Lipid extractions were performed and sphingosine was quantified by mass spectrometry. Data are means ± SD (*n = *3); *p<0.05, **p<0.01, ***p<0.001 compared to wt cells. (C) endogenous sphingosine in the wt carcinoma cell lines upon treatment with the SK-1 inhibitor SK I II (10 µM) or DMSO as vehicle control for 24 h. Quantification was done as above. Data are means ± SD (n = 3); *p<0.05, **p<0.01 compared to DMSO treated cells.

SK-1 regulates the sphingosine/S1P ratio and thus the commitment of cells to survival or death. We used carcinoma cell lines of different histotypes and with different SK-1 expression levels to stably downregulate SK-1 using shRNA, and measured the intracellular accumulation of sphingosine by mass spectrometry. As shown in Fig. 1A and Fig. S1A, in MDA-MB-231, NCI-H358, and HCT 116 cells vector-based RNAi strongly reduced SK-1 protein and mRNA expression (SK-1 kd cells) compared to the respective untreated wild-type (wt) cells. As further controls, MDA-MB-231 cells treated with empty virus or overexpressing SK-1 (SK-1 ov) were included, which showed no effect on SK-1 expression or strong overexpression, respectively. SK-2 expression was not modulated in SK-1 kd cells as shown in Fig. S1B. Interestingly, all SK-1 kd cells contained higher levels of sphingosine compared to their counterpart control cells and this increase was most pronounced in MDA-MB-231 SK-1 kd cells (Fig. 1B). Transfection of MDA-MB-231 cells with empty virus as additional control did not increase the level of sphingosine, indicating that they behaved like wt cells. On the other hand, sphingosine was reduced by 90% in MDA-MB-231 cells overexpressing SK-1 (SK-1 ov). The MDA-MB-231 cell line also expressed the highest basal level of SK-1, indicating that it was most susceptible to the manipulation and probably strongly depends on SK-1 function to keep the sphingolipid rheostat in check [26] **.** Ceramide levels were not altered in SK-1kd cells of all histotypes compared to wt (Fig. S1C). Similar to the genetic knockdown of SK-1, pharmacologic manipulation of SK-1 activity using the SK-1 inhibitor SKI II also increased sphingosine levels in the cell lines in correlation with their basal SK-1 expression (Fig. 1C).

### Elevated Sphingosine in SK-1 kd Cells Inhibits PKC

**Figure 2:**
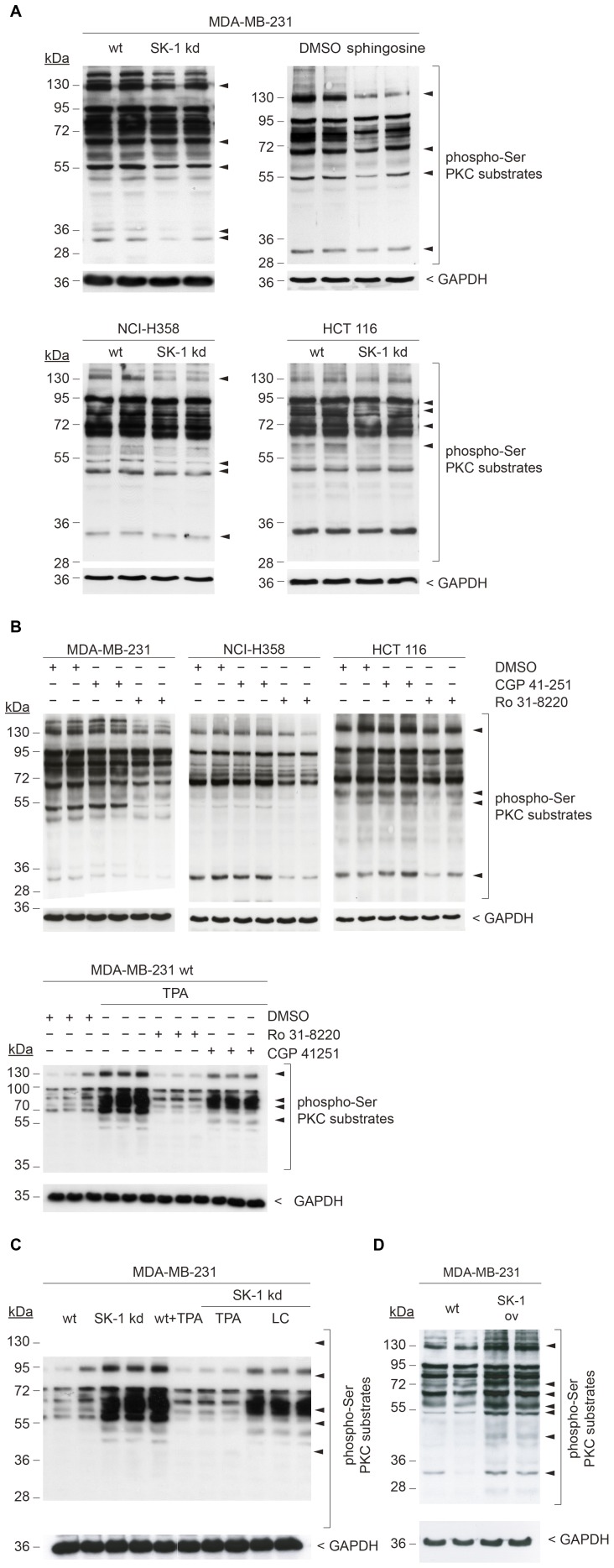
Downregulation of SK-1 increases sphingosine which inhibits PKC. (A) lysates were prepared from MDA-MB-231, NCI-H358, and HCT 116 wt and SK-1 kd cells, and analyzed for PKC activity by Western blotting using antibodies against various p(Ser) PKC substrates (dilution 1∶1000) or GAPDH (dilution 1∶2000) as loading control. MDA-MB-231 cells treated with sphingosine or DMSO vehicle control were analyzed for comparison. (B) cells were treated with the PKC inhibitors CGP 41-251 (300 nM) or Ro 31-8220 (1 µM) or with DMSO as control for 24 h before collection of lysates or quiescent MDA-MB-231 wt cells were pretreated for 4 h with the PKC inhibitors Ro 31-8220 (1 µM), CGP 41-251 (300 nM) or DMSO as control. Thereafter, cells were stimulated with the PKC activator TPA (200 nM) for 15 min. and lysates were analyzed for p(Ser) PKC substrates as described above. (C) quiescent MDA-MB-231 wt and SK-1 kd cells were stimulated with the PKC activator TPA (200 nM) for 15 min, treatment of SK-1 kd cells with lactacystin (20 µM for 24 h) was used to control for protein degradation. Lysates were analyzed for p(Ser) PKC substrates as described above. (D) MDA-MB-231 wt and SK-1 overexpressing (SK-1 ov) cells were analyzed for p(Ser) PKC substrates as described above.

Sphingosine can act as an endogenous inhibitor of PKC [27] and it was therefore of interest to investigate PKC activity in the three SK-1 kd carcinoma cell lines. Since direct measurement of PKC isoenzyme-specific activity is not reliable, we indirectly measured total PKC activity by detection of changes in the phosphorylation status of various isoenzyme-specific substrates. To this end, a phospho-Ser PKC substrate antibody was used which recognizes target proteins phosphorylated at the PKC consensus site R/K-X_−1_-S-X_+1_-R/K, where X_+1_ is a hydrophobic residue. In all SK-1 kd cell lines, a distinct subset of bands was reduced compared to the respective wt control cells (Fig. 2A). Specifically, reduced phosphorylation upon SK-1 downregulation was found for bands at 130, 80, 68, 55, and 36 kDa in MDA-MB-231 cells, at 130, 55, 50, and 34 kDa in NCI-H358 cells, and at 95, 80, 70, and 56 kDa in HCT 116 cells. The differences in the phosphorylation pattern and intensities among the different cell lines suggest that either different PKC isoenzymes were involved or that PKC isoenzymes and/or their substrates were differentially expressed. As shown for MDA-MB-231 cells, treatment with exogenous sphingosine (20 µM) resulted in a very similar pattern of reduced substrate phosphorylation compared to the SK-1 kd cells, thus confirming the inhibitory effect of this sphingolipid on PKC activity. In addition, we examined the levels of expression of different PKC isoenzymes in MDA-MB-231 wt, SK-1 kd and SK-1 ov cells and could not detect any differences (Fig. S2).

To better discriminate between the various PKC isoenzymes inactivated by sphingosine, the wt cell lines were treated with the inhibitor CGP 41-251, which targets the calcium-dependent PKC α, β, and γ isoenzymes [28], and the pan-PKC inhibitor Ro 31-8220, which targets all isoenzymes with the exception of PKCζ [29]. Fig. 2B shows that in all carcinoma cell lines Ro 31-8220, but not CPG 41251, effectively reduced phosphorylation of the PKC substrates. The same data were obtained under conditions of serum starvation of cells when pretreatment with Ro 31-8220 effectively prevented PKC from TPA activation, whereas CPG 41–251 was only partially effective (Fig. 2B). This finding identified calcium-independent PKC isoenzymes as the major targets inhibited in SK-1 kd cells.

As a reverse approach we stimulated MDA-MB-231 wt and SK-1 kd cells with phorbol ester (TPA) as an activator of PKC [14], [28] and examined which PKC substrates became phosphorylated. As shown in Fig. 2C, stimulation with TPA increased the phosphorylation in a number of bands with the most pronounced alterations occurring at the same size (130, 80, 55, 36 kDa) where phosphorylation was decreased in SK-1 kd cells and in wt cells upon treatment with exogenous sphingosine (Fig. 2A). This confirms that these bands indeed represent relevant PKC substrates. Similar results were obtained with wt cells in the presence of the SK-1 inhibitor SK I II (data not shown). Furthermore, blocking protein degradation in SK-1 kd cells using lactacystin (LC) failed to restore substrate phosphorylation (Fig. 2C), indicating that reduced phosphorylation was not due to substrate degradation but rather to inhibition of PKC and/or decreased PKC substrate expression.

Inversely, in MDA-MB-231 cells overexpressing SK-1 phosphorylation of the same PKC substrates which were inhibited in SK-1 kd cells was increased compared to wt cells (Fig. 2D). This further confirms the ability of SK-1 to stabilize PKC activity, probably by depletion of intracellular sphingosine.

### Reduced Colony Formation of SK-1 kd Cells can be Mimicked with Sphingosine or PKC Inhibitors

**Figure 3:**
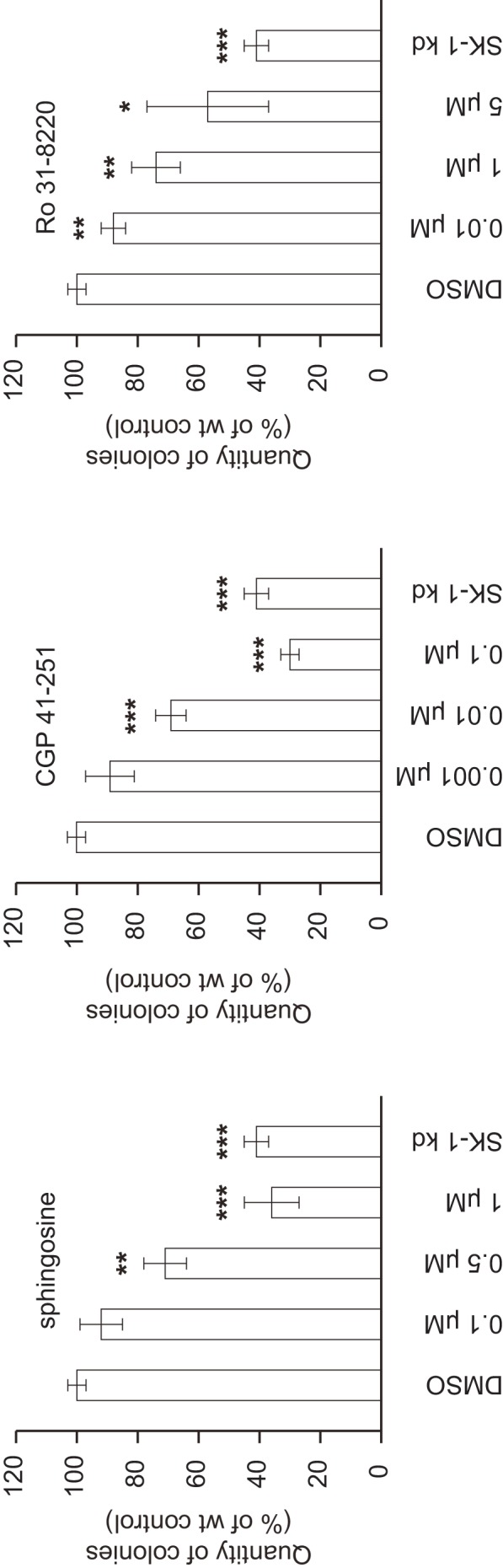
Inhibition of PKC decreases colony formation of carcinoma cells. MDA-MB-231 wt cells were seeded in 60 mm diameter dishes at a density of 700 cells per dish in cell culture medium. After 24 h cells were treated with various concentrations of the PKC inhibitors CGP 41-251, Ro 31-8220 or with sphingosine, DMSO was used as vehicle control. Colony formation of SK-1 kd cells (untreated) incubated under identical conditions is shown for comparison. Cells were incubated for another 14 d before colonies were stained with 2% crystal violet and counted using a ColCountTM (Mammalian Cell Colony Counter, Oxford Optronix). Only colonies containing more than 50 cells were evaluated. Data are means ± S.D. (n = 3); *p<0.05, **p<0.01, ***p<0.001 compared to the DMSO control values.

The cellular effects associated with PKC inhibition in tumor cells include decreased proliferation, cell cycle disruption and apoptosis [30]. Since downregulation of SK-1 increases intracellular sphingosine, which inhibits PKC and decreases cell proliferation and survival, the colony formation of MDA-MB-231 wt cells upon treatment with sphingosine or the two PKC inhibitors was measured and compared to SK-1 kd cells. As shown in Fig. 3, treatment with either sphingosine, CGP 41-251 or Ro 31-8220 reduced colony formation in a dose-dependent manner. CGP 41-251 more effectively attenuated clonal expansion than Ro 31-8820, which might be due to its negative effect on other kinases involved in cell proliferation [31], [32] **.** Compared to SK-1 kd cells a similar degree of inhibition was achieved with >0.5 µM sphingosine, 0.01 µM CGP 41-251 or with 5 µM Ro 31-8220. From this we conclude that intracellular sphingosine and PKC are key players in controlling the clonal expansion of carcinoma cells.

### SK-1 kd Cells Enter Mitosis but Fail to Divide

**Figure 4:**
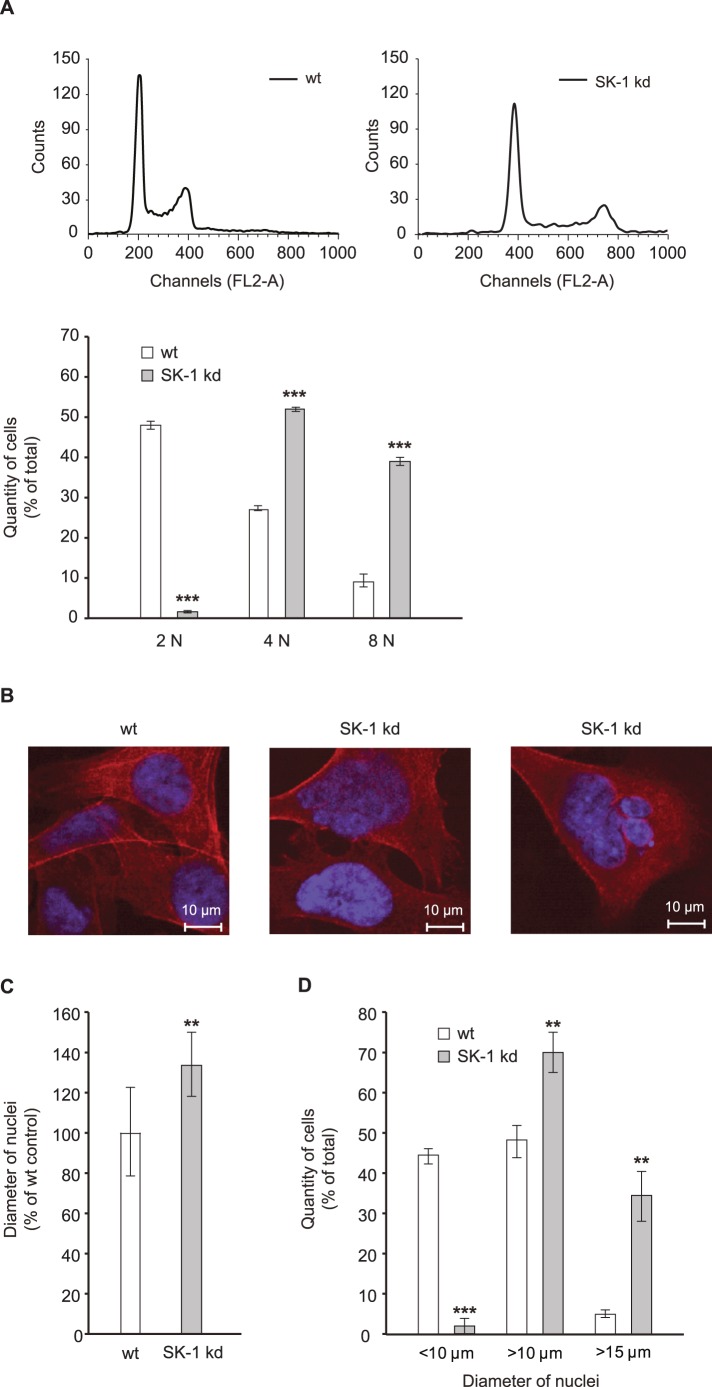
Downregulation of SK-1 induces endoreduplication. (A) MDA-MB-231 wt cells and SK-1 kd cells were stained with propidium iodide and DNA content was measured by flow cytometry using a FACSCalibur flow cytometer and the Cell Quest software for data processing. (B) confocal microscopy of MDA-MB-231 wt and SK-1 kd cells upon actin (red) and nuclear staining (blue) using TRITC-labeled phalloidin (dilution 1∶1000) and DAPI, respectively. (C) calculation of the nuclear size of SK-1 kd cells relative to wt cells. (D) quantification of cells with nuclei in different size ranges (<10 µM, >10 µM, >15 µM). Data are means ± SD of three independent experiments; **p<0.01, ***p<0.001 compared to MDA-MB-231 wt cells values.

Sphingolipids and PKC are implicated in the control of cytokinesis and mitotic exit [33], [34]. To investigate whether the decreased proliferation and clonal expansion of MDA-MB-231 SK-1 kd and of wt cells upon treatment with sphingosine or PKC inhibitors was due to mitotic defects and cytokinetic regression, the DNA content was measured. Compared to wt cells there was an almost complete lack of SK-1 kd cells with a 2N DNA content whereas the fraction of cells with 4N and 8N was substantially increased (Fig. 4A). The data of co-treatment with SK I II inhibitor and sphingosine shown in Fig. S3A also suggest that sphingosine and inhibition of SK-1 are responsible for the accumulation of cells in S phase and with 4N and 8N DNA content after 24 h of treatment. Moreover, after 48 h and 72 hours of treatment we observed an accumulation of cells in the SubG1 peak, indicating increased apoptosis (Fig. S3A, Fig. S3B). As shown in Fig. 4B, cytokinesis failure in MDA-MB-231 SK-1 kd cells was also confirmed by an increased number of giant cells with substantially enlarged nuclei or multinucleation compared to wt cells. The diameter of the SK-1 kd cells was increased by 30% (Fig. 4C) and nuclei with diameter less than 10 µM were almost absent in kd cells (Fig. 4D). Quantification of phospho-histone H3 revealed no decrease in the overall number of mitotic cells (data not shown).

By tendency, short term treatment with sphingosine, the SK-1 inhibitor SK I II, the PKC inhibitor Ro 31-8220, or with SK I II and sphingosine simultaneously (Fig. S3A) or sphingosine alone (data not shown) also increased endoreduplication in MDA-MB-231 wt cells (data not shown). In contrast to genetic manipulation of cells with shRNA where SK-1 was permanently decreased and cells were allowed to adapt over time, treatment with the pharmacologic inhibitors resulted in substantial toxicity thereby precluding selection of a stable population.

### SK-1 Downregulation Compromises cdc2 and Chk1 Function

**Figure 5:**
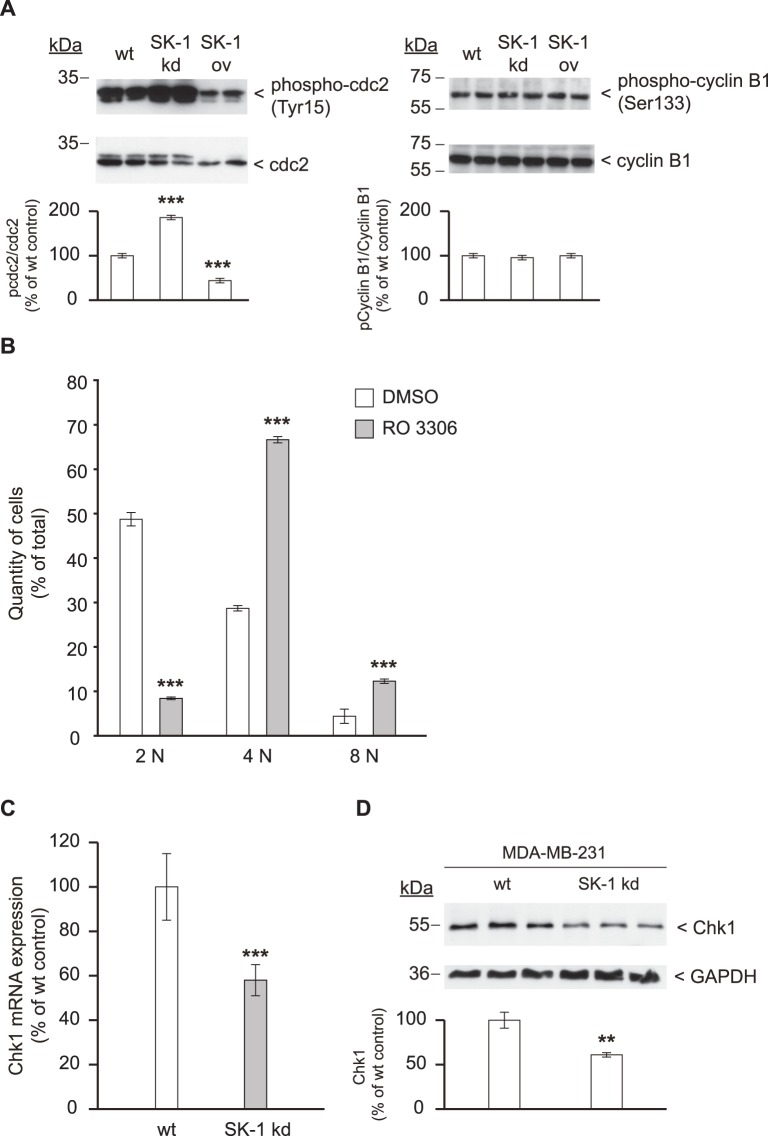
Downregulation of SK-1 inhibits cdc2 activity and decreases Chk1 expression. (A) lysates from MDA-MB-231 wt, SK-1 kd, and SK-1 overexpressing cells (SK-1 ov) were subjected to Western blotting using antibodies against total cdc2 (dilution 1∶2000), phospho-cdc2(Tyr15) (dilution 1∶1000), total cyclin B1, and phospho-cyclin B1(Ser133) (dilution 1∶1000). The films were digitized and for each protein lane a density blot was measured. Each value in the graph represents the mean ± SD band density for each group (*n = *3); ***p<0.001 compared to wt cells (B) MDA-MB-231 wt cells were treated with the cdc2 inhibitor RO 3306 (9 µM) or DMSO as vehicle control for 24 h, stained with propidium iodide and DNA content was measured as described in Fig. 4. (C) Chk1 mRNA expression in MDA-MB-231 wt and SK-1 kd cells was measured by quantitative real-time PCR and data were normalized to 18S rRNA. ***p<0.001 compared to wt values. (D) Chk1 protein expression in MDA-MB-231 wt and SK-1 kd cells detected by Western blotting using antibodies against Chk1 (dilution 1∶500) or GAPDH (dilution 1∶2000) as loading control. The films were digitized and for each protein lane a density plot was measured. Each value in the graph represents the mean ± SD band density for each group (*n = *3); **p<0.01 compared to wt cells.

To understand the molecular mechanism underlying endoreduplication and cytokinesis failure in MDA-MB-231 SK-1 kd cells, we examined the expression and phosphorylation of cyclin B1 and cdc2 by Western blotting. As shown in Fig. 5A, there was no increase in the level of total cdc2, cyclin B1, and phospho-cyclin B1(Ser133) in SK-1 kd cells compared to MDA-MB-231 wt cells. However, we found a 2-fold increase in the inhibitory phosphorylation of cdc2(Tyr15) compared to wt cells and a 4-fold increase in the inhibitory phosphorylation of cdc2(Tyr15) compared to SK-1 ov cells. Inversely, Tyr15 phosphorylation was reduced 4-fold in SK-1 ov cells compared to SK-1 kd cells and 2-fold compared to wt cells.

Decreased cdc2 activity may compromise cell cycle progression and cytokinesis as recently demonstrated using the cdc2-specific inhibitor RO 3306 [22]. Indeed, as shown in Fig. 5B treatment of MDA-MB-231 wt cells with RO 3306 also induced a massive loss of cells with 2N DNA content and concomitantly increased the number of 4N and >4N cells, resulting in a profile reminiscent of SK-1 kd cells.

Furthermore, we examined expression of the checkpoint kinase Chk1, which is essential during mitosis and cytokinesis to complete cell division. Figs. 5C and 5D show that in SK-1 kd cells Chk1 expression was indeed decreased on the mRNA and protein level compared to wt cells. This suggests that the mitotic and cytokinetic defects of SK-1 kd cells resulted from the combined loss of cdc2 and Chk1 function.

### SK1 Downregulation Compromises Spindle Checkpoint Function

**Figure 6:**
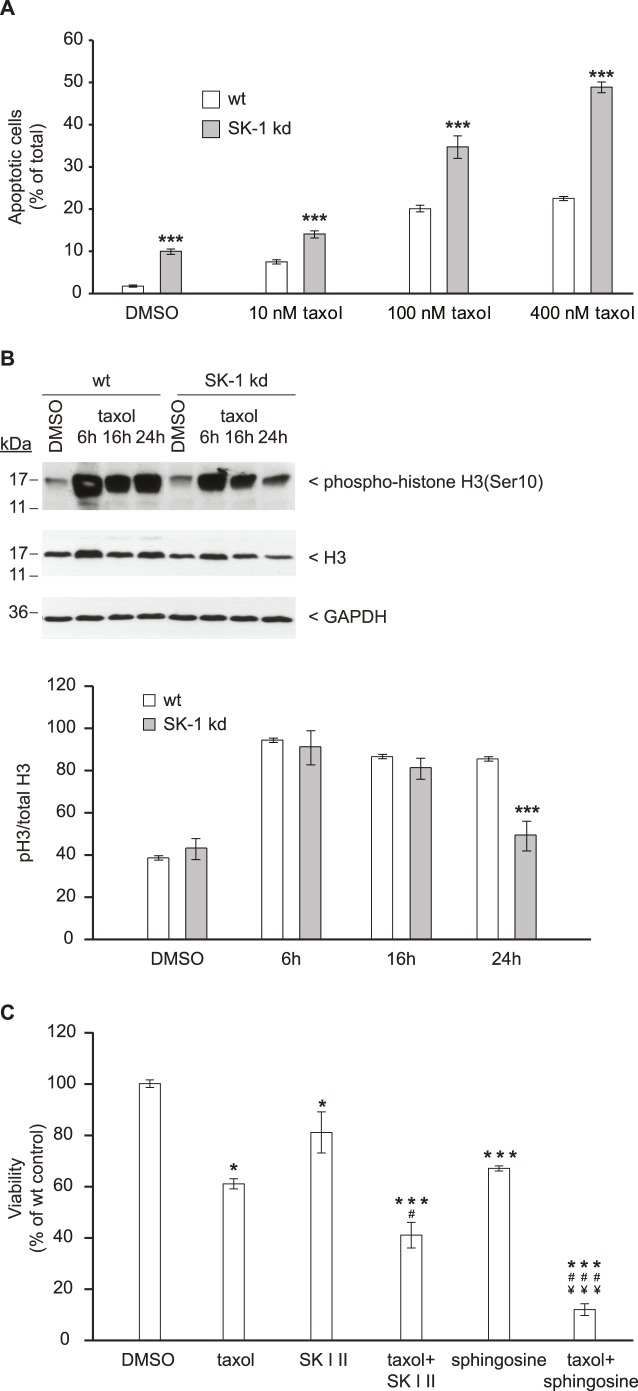
Downregulation of SK-1 compromises M phase arrest and sensitizes cells to taxol. (A) MDA-MB-231 wt and SK-1 kd cells were treated with various concentrations of taxol or DMSO as vehicle control for 24 h before apoptosis was measured by annexin V staining using flow cytometry. Data are means ± SD (n = 3); ***p<0.001 compared to DMSO treated cells. (B)**,** detection of phospho-histone H3(Ser10) as a marker of the mitotic index in MDA-MB-231 wt and SK-1 kd cells at different time points upon treatment with taxol (100 nM). Lysates were subjected to Western blotting using antibodies against total histone H3 (dilution 1∶2000) and phospho-histone H3(Ser10) (dilution 1∶1000). The Density of phospho-histone H3(Ser10) (pH3)/H3 ratio in taxol-treated MDA-MB-231 cells is presented as % of total H3. Each value in the graph represents the mean ± SD band density for each group (*n = *3); ***p<0.001 compared to wt cells. (C) MDA-MB-231 wt cells were treated with taxol (100 nM), the SK-1 inhibitor SK I II (10 µM), sphingosine (20 µM) or the various combinations for 48 h. DMSO was used as vehicle control. Cell viability was determined in colorimetric MTT assays. Data are means ± S.D. (n = 3); *p<0.05, ***p<0.001 compared to DMSO treated cells; ^#^p<0.05, ^###^p<0.001 compared to the respective taxol treated cells; ^¥¥¥^p<0.001 compared to the respective SK I II treated cells.

Chk1 expression is reduced in SK-1 kd cells, which may compromise spindle checkpoint function [35] and thus sensitize cells to spindle-targeted chemotherapy. To test this possibility, MDA-MB-231 wt and SK-1 kd cells were treated with taxol and apoptosis was measured by annexin V staining. The data depicted in Fig. 6A confirm that SK-1 kd cells were clearly more sensitive to taxol-induced apoptosis than their wt counterparts. This effect was independent of ceramide, which was not increased in SK-1 kd cells (data not shown). Further analysis revealed that 24 h upon taxol treatment the level of phospho-histone H3 as a mitotic marker decreased (Fig. 6B), indicating that less SK-1 kd cells accumulated in the M phase compared to wt cells and that their mitotic exit was enforced.

In addition, in a complementary viability assay we investigated the ability of sphingosine and the SK-1 inhibitor SK I II to sensitize MDA-MB-231 wt cells to taxol. As shown in Fig. 6C, combining taxol and SK I II resulted in an additive cytotoxic effect on cells, which was even more than additive for the combination of taxol and sphingosine.

These findings confirm that targeting SK-1 in carcinoma cells generates an unfavorable mitotic environment with the potential to increase the efficacy of taxane-based chemotherapy.

## Discussion

We demonstrate that downregulation of SK-1 in various carcinoma cell lines leads to intracellular accumulation of sphingosine, a bioactive lipid with proapoptotic potential, whereas the level of ceramide was not affected. This effect was most pronounced in MDA-MB-231 cells. Since these cells exhibited the highest basal level of SK-1, one might speculate that this is necessary to keep the amount of the proapoptotic substrate in check. The relative concentrations of the sphingolipid metabolites S1P, sphingosine, and ceramide maintain a rheostat implicated in cell death and survival [2]. As shown previously, SK-1 knockdown in carcinoma cells not only increases intracellular sphingosine but conversely also depletes the pool of prosurvival S1P, resulting in growth inhibition and apoptosis [9], [26]. In contrast to sphingosine, S1P cannot enter into cells. Although it also acts as a survival factor by binding to cognate receptors on the cell surface, this is independent of the biological effect of intracellular S1P.

Interestingly, we found that in all three SK-1 kd carcinoma cell lines accumulation of sphingosine decreased PKC activity and this effect could be mimicked by the addition of exogenous sphingosine to cells. On the other hand, PKC activity was increased in cells overexpressing SK-1. The ability of sphingosine to inhibit PKC by interaction with its regulatory domain was first described by Hannun et al. [36] and later studies demonstrated a direct competition of sphingosine with phorbol ester binding to PKC [11]. However, so far the biological relevance of the SK1-PKC connection has not been investigated in the context of SK-1-targeted cancer therapy. PKC enzymes are major players in the regulation of cell proliferation and survival with distinct biological roles and cell type specificities. Despite many years of intensive research in the field, the implication of its various isoforms in cancer is still unclear [30]. Here, we measured Ser phosphorylation of various PKC substrates as a common approach to determine cellular PKC activity [37], [38]. Although we could not fully dissect the distinct PKC isoforms inhibited by sphingosine in our carcinoma cells, using selective inhibitors we excluded α and β, and identified Ca^2+^-independent isoforms as major targets. The effect of CGP 41-251 on colony formation without detectable activity in the p-Ser-PKC substrate assay is likely due to inhibition of other survival kinases required for cell proliferation [31], [32]. Taking the plethora of biological effects of PKC into account, we conclude that SK-1 owes its oncogenic potential to the increased production of prosurvival S1P, which at the same time prevents accumulation of the proapoptotic substrate sphingosine and its detrimental effect on PKC. Thus, SK-1 connects to PKC-controlled cell survival pathways by regulating the intracellular level of sphingosine as a biological PKC inhibitor. Indeed, in terms of proliferation, colony formation and overall viability SK-1 kd cells behaved similar to cells treated with PKC inhibitors [37], [39], [40]. Conversely, PKC can act as an upstream regulator of SK-1 as it was shown that in HEK cells phorbol ester not only activated PKC but also SK-1, and inhibition of PKC on the other hand impaired SK-1 function by preventing its translocation to the plasma membrane [41]. Similarly, in renal mesangial cells inhibition of PKC also decreased SK-1 expression and attenuated its activation by growth factors [42].

From all three carcinoma cell lines we established sublines in which SK-1 was stably downregulated by vector-based RNAi. Alternatively, we used a pharmacologic SK-1 inhibitor SK I II [5] to measure the short term effects of SK-1 loss. In both systems, the level of intracellular sphingosine increased compared to untreated cells. Not unexpectedly, differences in the effect of the two SK-1 targeting strategies were observed only in terms of the strength of the cell response, but not by tendency. This was probably due to selection of shRNA-transduced cells which had adapted to survive without SK-1, whereas in contrast, selection of a stable SK-1^low^ population using the SK I II inhibitor failed due to increasing toxicity over time.

Apart from the loss of PKC activity the SK-1 kd carcinoma cells may contain less S1P, a survival factor maintaining constant cross-talk with other major growth factors [43], [44]. Recently, SK-1 was found to localize to centrosomes together with the S1P_5_ receptor [7]. Whether S1P is produced in the nucleus and whether its mitogenic activity can directly affect the cell cycle machinery remains to be demonstrated. Here, we show that in MDA-MB-231 cells, which showed the strongest response to SK-1 downregulation in terms of sphingosine generation and PKC inhibition, loss of SK-1 was accompanied by endoreduplication and the formation of giant cells with enlarged nuclei or multinucleation. Typically, these cells showed mitotic defects comprising spindle checkpoint and cytokinesis failure reminiscent of cells in which other mitosis regulators such as Aurora B kinase are compromised [45]. However, we did not find evidence for the modulation of Aurora B in SK-1 kd cells (Fig. S4). Again, similar but weaker effects were observed upon short-term treatment with sphingosine or the PKC inhibitor RO 31-8220, which for reasons of toxicity could not be used in long-term assays to select a viable and stable SK-1^low^ cell population. However, despite an increasing number of cells with 4N DNA content, we could not find signs of increased endoreduplication. The ability of sphingosine and staurosporine to induce G2/M arrest was also described for other cell types [10], [46]. One might thus speculate that this was a transitional phenomenon reflecting early effects of PKC inhibition in this cell population. Our molecular analysis unveiled that the activity of cdc2, one of the components of the M phase promoting factor (MPF) required to enter mitosis and prevent premature exit without completion of cytokinesis [21], [22], [47], was decreased in MDA-MB-231 cells upon SK-1 downregulation or sphingosine treatment. Conversely, cdc2 activity was increased in cells overexpressing SK-1. It is possible that the decreased cdc2 activity in SK-1 kd cells was due to inhibition of PKC, which was previously shown to activate the MPF [23].

In addition to decreased cdc2 activity, expression of Chk1 was also reduced in MDA-MB-231 SK-1 kd cells. Since Chk1 delays entry of cells with damaged or unreplicated DNA into mitosis [48], one might expect that SK-1 kd cells are more susceptible to the lethal effect of DNA damage. In fact, the rate of apoptosis in SK-1 cells was moderate in the absence of cytotoxic treatment and analysis of Beclin 1 (Atg 6) expression did not provide evidence for increased autophagy (Fig. S4). The cells could be propagated, albeit with a slower growth rate than wt cells. Since MDA-MB-231 cells lack functional p53, they have a deficient post-mitotic checkpoint and also processes of depolyploidization might be involved which limit the number of cells undergoing cell death by mitotic catastrophe [49]. On the other hand, we previously found that SK-1 knockdown in carcinoma cells facilitates apoptosis induction by DNA damage [26]. Recently, reduced expression of Chk1 and consequently sensitization to ionizing radiation was described in cells depleted for S1P by transfection of S1P lyase [50]. It is thus tempting to speculate that S1P somehow connects to the cell cycle machinery via Chk1. Even more relevant for the present study is that Chk1 is also required for spindle checkpoint function [24], [25], and its loss of function may cause premature mitotic entry followed by metaphase block, misalignment of chromosomes, kinetochore defects, and cytokinetic regression [25], [35]. Therefore, in MDA-MB-231 SK-1 kd cells the impaired function of the two M phase regulators cdc2 and Chk1 together likely aggravates mitotic defects and reduces cell viability, particularly upon spindle-targeted chemotherapy. Evidence for such a lethal cooperation was also provided by the increased apoptosis of SK-1 kd cells upon treatment with taxol, and the more than additive cytotoxic effect when the PKC inhibitor sphingosine and the SK-1 inhibitor were combined with taxol. Facilitation of apoptosis was independent of ceramide, which did not increase upon SK-1 downregulation.

Altogether, our data demonstrate that downregulation of SK-1 in carcinoma cells leads to intracellular accumulation of sphingosine which inhibits PKC. Inhibition of PKC in SK-1 kd cells reduced proliferation and viability, and was accompanied by decreased cdc2 and Chk1 function, which compromised spindle checkpoint function and cytokinesis. More investigations are warranted to further delineate the intriguing connection between SK-1, PKC and the mitosis machinery.

## Materials and Methods

### Antibodies and Reagents

Hyperfilm MP, nitrocellulose membranes, secondary horseradish peroxidase-coupled IgG, and enhanced chemiluminescence reagents were obtained from GE Health Care Systems GmbH (Glattbrugg, Switzerland). 2-(*p*-Hydroxyanilino)-4-(*p*-chlorophenyl) thiazole (SKI II), Taxol (paclitaxel), Aurora B antibody and TRITC-labeled phalloidin for actin staining were from Sigma-Aldrich (Buchs, Switzerland). Antibodies against phospho-Ser–PKC substrate, PKD/PKC µ, phospho-cdc2(Tyr15), total cdc2, phospho-cyclin B1(Ser133), total cyclin B1, total Histone H3 and GAPDH were obtained from Cell Signaling (supplied by Bioconcept, Allschwil, Switzerland). The antibodies against PKC Θ, λ, ι, ζ were from Transduction Laboratories (San Diego, California). Synthetic peptides based on the C-terminal sequence of PKC α (SYVNPQFVHPILQSAV), PKC ε (NQEEFKGFSYFGEDLMP) and PKC δ (KGFSFVNPKYEQFLE) were synthesized on an ARI 431 peptide synthesizer, coupled to keyhole limpet hemocyanin by glutaraldehyde and used to immunize rabbits. The detailed characterization of anti- PKC α, ε, δ antibodies is described elsewhere [28], [51]–[54]. The antibody against phospho-histone H3(Ser10) was from Millipore (supplied by Lubioscience, Luzern, Switzerland). Antibodies against Beclin 1 (Atg 6) and Chk1, D-erythro-sphingosine and the PKC inhibitor Ro 31-8220 were obtained from Santa Cruz Biotechnology (supplied by LabForce AG, Nunningen, Switzerland). The antibody against human SK-1 was generated as described [55]. To generate the polyclonal anti-human SK-2 antibody, two synthetic peptides, NGHLEAEEQQDQRPD and CLPGDGEITPDLLPRP based on the sequence of human SK-2 (accession number: NM_020126) were synthesized and coupled to keyhole-limpet hemocyanin, and used to immunize rabbits. The antiserum denoted #65 was characterized using transiently human SK-2 overexpressing HEK293 cell lysates. Western blot analysis revealed one single band at 68 kDa. Recombinant GFP-labeled annexin V was kindly provided by Dr. T. Kaufmann (Institute of Pharmacology, Bern). Glycergel Dako mounting medium was supplied by DiaLine AG (Lausen, Switzerland). Lactacystin, PKC inhibitor CGP 41-251 and the cdc2 inhibitor RO-3306 were from Merck Biosciences (Darmstadt, Germany).

### Carcinoma Cell Lines and Cell Culture

Cell culture media, serum and supplements were purchased from Invitrogen AG (Basel, Switzerland). The breast carcinoma cell line MDA-MB-231 was obtained from the European Collection of Cell Cultures (ECACC) through Sigma-Aldrich, NCI-H358 lung and HCT 116 colon carcinoma cells were obtained from American Type Culture Collection (ATCC, Manassas, VA, USA). MDA-MB-231 and NCI-H358 were cultured in RPMI medium supplemented with 10% fetal bovine serum, 10 mM Hepes pH 7.4, and 100 units/ml each of penicillin and streptomycin. HCT 116 cells were cultured in McCoy medium containing 10% fetal bovine serum, 10 mM Hepes pH 7.4 and 100 units/ml each of penicillin and streptomycin. All cells were grown at 37°C in a humidified atmosphere containing 5% CO_2_.

SK-1 downregulation in cells was performed using the All MISSION^R^ shRNA constructs from Sigma-Aldrich as described [26]. SK-1 knockdown cells (SK-1 kd) were selected in medium containing 1 µg/ml puromycin (Santa Cruz Biotechnology). SK-1 overexpressing MDA-MB-231 cells (SK-1 ov) were generated by stable transfection of the full length SK-1 cDNA cloned into pcDNA3.1 (Invitrogen AG). Transfection was performed as recommended by the supplier.

### Sphingolipid Quantification by LC/MS/MS

Equal numbers of cells in 6-well plates were scraped in methanol containing internal C17-S1P and C17-sphingosine, C17-ceramide standards and subjected to lipid extraction and mass spectrometry as described [56].

### Quantitative Real-time PCR

The expression of Chk1, SK-1 mRNA was quantified using a BioRad iQ5-Cycler Detection System (Bio-Rad Laboratories AG, Reinach, Switzerland). The following primer sequences were used: human Chk1 forward: AAG CAG TCG CAG TGA AGA TTG TAG, reverse: TTG CCT TCT CTC CTG TGA CCA TAG; human SK-1 forward: ACG CTC TGG TGG TCA TGT CTG, reverse: AGT TGG TCA GGA GGT CTT CAT TGG TTG CCT TCT CTC CTG TGA CCA TAG; 18 S rRNA forward: CGA TTC CGT GGG TGG TG GTG, reverse: CAT GCC AGA GTC TCG TTC GTT ATC. The IQTM5 Optical System Software (Version 2.0, Bio-Rad) was used to analyze real time and endpoint fluorescence.

### Western Blotting

Cell homogenization, sample preparation and Western blotting was performed as described [26] using the indicated antibodies.

### Cell Viability Assay

Cell viability was measured using the MTT cell proliferation kit (Roche Diagnostics AG, Rotkreuz, Switzerland) as described by the manufacturer. Briefly, 5×10^4^ cells were seeded in 96-well plates and allowed to adhere overnight. Thereafter, cells were treated for 48 h with the indicated inhibitors or vehicle (DMSO) before the MTT reagent was added for 5 h and the absorbance of formazan was measured using a SpectraMax M2 microplate reader (Molecular Devices, Bucher Biotech AG, Basel, Switzerland).

### Colony Formation Assay

Cells were cultured in 60 mm diameter dishes at a density of 700 cells per dish in cell culture medium. After 24 h, cells were treated with CGP 41-251, Ro 31-8220, sphingosine or DMSO as vehicle control, and incubated for 14 days to allow colony formation. Then, cells were washed with ice-cold PBS, air dried, stained for 30 min with 2% crystal violet, washed with water, and air dried again. The number of colonies was counted using a ColCountTM (Mammalian Cell Colony Counter, Oxford Optronix, Oxford, U.K.). Only colonies containing more than 50 cells were evaluated.

### Cell Cycle Analysis

Cells were harvested by trypsinization, washed and fixed in 70% ethanol overnight at -20°C. Thirty minutes prior to analysis, cells were resuspended in PBS containing 50 µg/ml propidium iodide (PI) and 5 µg/ml RNase A. Samples were analyzed using a FACSCalibur flow cytometer and the Cell Quest software (both from Becton Dickinson Biosciences, Allschwil, Switzerland). In each sample, 10′000 cells sorted for red fluorescence were analyzed.

### Confocal Microscopy

To analyze nuclear morphology, cells were grown on 12 mm glass cover slips (BD Biosciences), fixed with 4% paraformaldehyde and stained for actin using TRITC-labeled phalloidin and DAPI for nuclei. The anti-fading fluorescent mounting medium (Dako) was added and slides were covered with coverslips and analyzed by confocal laser scanning microscopy (LSM 5 Exciter, Carl Zeiss MicroImaging GmbH, Jena, Germany). Quantification of cells and measurement of the largest diameter of nuclei was performed using a Zeiss LSM Image Browser (Carl Zeiss MicroImaging GmbH). At least 30 cells in the field were counted and examined by three independent determinations.

### Apoptosis Assay

The fraction of cells with fragmented DNA was quantified from the SubG1 peak as described under cell cycle analysis. In addition, apoptotic cells were detected by PI staining and surface binding of annexin V. To this end, cells were harvested by trypsinization, washed and resuspended at a concentration of 6×10^5^ cells/ml in binding buffer containing 150 mM NaCl, 4 mM KCl, 2.5 mM CaCl_2_, 1 mM MgSO_4_, 15 mM HEPES. Cells were incubated with GFP-coupled annexin V (dilution 1∶1000) and PI (dilution 1∶500), analyzed using a FACSCalibur flow cytometer and the Cell Quest software (Becton Dickinson Biosciences).

### Statistics

Statistical analysis of data was performed with Graph Pad Prism 4 (Graph Pad Software Inc., La Jolla, CA, USA). Results are given as means ± SD. For two-group analyses Student’s unpaired two-tailed *t*-test was used. Statistical analyses of multigroup data were performed by one way analysis of variance (ANOVA) with Bonferroni correction. p<0.05 was considered statistically significant.

## Supporting Information

Figure S1 **Transduction of carcinoma cell lines with SK-1 shRNA decreases SK-1 mRNA expression.** (A) MDA-MB-231, NCI-H358, and HCT 116 cells were transduced with lentiviral SK-1 shRNA to downregulate SK-1 expression (SK-1 kd) or left untreated (wild-type, wt). SK-1 mRNA was measured by quantitative real-time PCR and data were normalized to 18S rRNA. Data are means ± SD (n = 3); **p<0.01, ***p<0.001, compared to wt cells. (B) lysates were prepared from MDA-MB-231, NCI-H358, and HCT 116 wt and SK-1 kd cells, and analyzed for SK-2 by Western blotting using antibody against SK-2 (dilution 1∶1000) or GAPDH (dilution 1∶2000) as loading control. (C) cellular ceramide was determined in the genetically modified SK-1 kd carcinoma cell lines, in the respective wt cells. Lipid extractions were performed and sphingosine was quantified by mass spectrometry. Data are means ± SD. (C) endogenous sphingosine in the wt carcinoma cell lines upon treatment with the SK-1 inhibitor SK I II (10 µM) or DMSO as vehicle control for 24 h. Quantification was done as above. Data are means ± SD (n = 3); *p<0.05, **p<0.01 compared to DMSO treated cells.
(EPS)

Figure S2 **PKC isoenzyme expression in MDA-MB-231 cells.** Lysates were prepared from MDA-MB-231 wt, SK-1 kd and SK-1 ov cells, and analyzed for PKC isoenzymes by Western blotting using specific antibodies against the various PKC proteins (dilution 1∶1000) or GAPDH (dilution 1∶2000) as loading control.
(EPS)

Figure S3 **Inhibition of SK-1 and sphingosine treatment increases cells in SubG1 and with 4N and 8N DNA content.** (A) MDA-MB-231 wt cells were seeded in 60 mm diameter dishes at a density of 3×105 cells per dish in cell culture medium. After 24 h, cells were treated with combination of SK I II inhibitor and sphingosine, DMSO was used as vehicle control. After another 24 h, cells were fixed, stained with propidium iodide and the DNA content was measured by flow cytometry using a FACSCalibur flow cytometer and the Cell Quest software for data processing. Data are means ± S.D. (n = 3); *p<0.05, ***p<0.001 compared to the DMSO control values. (B) MDA-MB-231 wt cells were seeded in 60 mm diameter dishes at a density of 3×105 cells per dish in cell culture medium. After 24 h, cells were treated with the SK I II inhibitor or sphingosine, DMSO was used as vehicle control. After another 24 h and 72 h, cells were fixed, stained with propidium iodide and the DNA content was measured by flow cytometry using a FACSCalibur flow cytometer and the Cell Quest software for data processing. Data are means ± S.D. (n = 3); *p<0.05, **p<0.01, ***p<0.001 compared to the DMSO control values.
(EPS)

Figure S4 **Downregulation of SK-1 in MDA-MB-231 cells does not affect expression of Beclin 1 and Aurora B.** Lysates were prepared from MDA-MB-231 wt and SK-1 kd cells, and analyzed for Beclin 1 (Atg 6) (dilution 1∶1000), Aurora B (dilution 1∶1000) and GAPDH (dilution 1∶2000) as loading control by Western blotting using specific antibodies.
(EPS)
