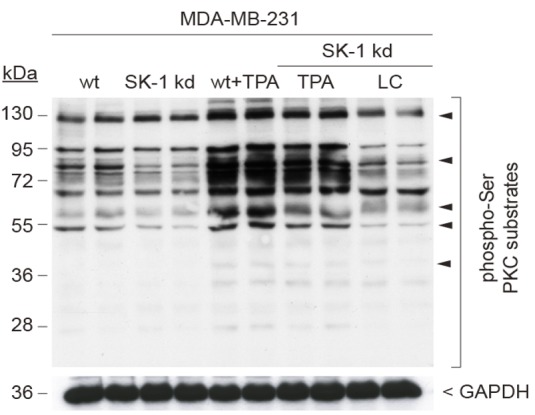# Correction: Targeting Sphingosine Kinase 1 in Carcinoma Cells Decreases Proliferation and Survival by Compromising PKC Activity and Cytokinesis

**DOI:** 10.1371/annotation/cf556c9c-8c18-4d54-b34f-7a2e0c9339e3

**Published:** 2013-10-29

**Authors:** Nataliya Kotelevets, Doriano Fabbro, Andrea Huwiler, Uwe Zangemeister-Wittke

The image in Figure 2C is unreadable. The correct version of Figure 2C can be viewed here: 

**Figure:**